# Coevolution between the cost of decision and the strategy contributes to the evolution of cooperation

**DOI:** 10.1038/s41598-019-41073-9

**Published:** 2019-03-14

**Authors:** Tetsushi Ohdaira

**Affiliations:** Institute of Information and Media, Aoyama Gakuin University, 5-10-1 Fuchinobe, Chuo-ku, Sagamihara-city, Kanagawa 252-5258 Japan

## Abstract

Cooperation is still an important issue for both evolutionary and social scientists. There are some remarkable methods for sustaining cooperation. On the other hand, various studies discuss whether human deliberative behaviour promotes or inhibits cooperation. As those studies of human behaviour develop, in the study of evolutionary game theory, models considering deliberative behaviour of game players are increasing. Based on that trend, the author considers that decision of a person requires certain time and imposes a psychological burden on him/her and defines such burden as the cost of decision. This study utilizes the model of evolutionary game theory that each player plays the spatial prisoner**’**s dilemma game with opponent players connected to him/her and introduces the cost of decision. The main result of this study is that the introduction of the cost of decision contributes to the evolution of cooperation, although there are some differences in the extent of its contribution regarding the three types of sparse topology of connections. Regarding the distribution of the cost of decision, especially in the case of the scale-free topology of connections, players with high cost of decision, which seem to be disadvantageous at first glance, sometimes become mainstream at the last.

## Introduction

Cooperation remains an important puzzle to both evolutionary and social scientists. Methods for sustaining cooperation that have received considerable attention involve costly punishment, social exclusion, resource allocation, feedback-evolving game, and so on. For example, regarding costly punishment, Chen *et al*.^1^ consider probabilistic punishment as the simplest way of distributing the responsibility to sanction defectors. Another study^2^ introduces class-specific probability of punishment that is based on the fixed number of classes. Regarding social exclusion (another form of punishment), Liu *et al*.^3^ find that such exclusion can always outperform punishment both in the optional and in the compulsory public goods game. Regarding resource allocation, Wang *et al*.^4^ introduce the regime that when contributing resources in the common pool exceed the threshold, the first divided resources will be equally allocated by all players, and the second divided ones will be allocated by all players based on their strategy choices. Finally, regarding feedback-evolving game, Chen and Szolnoki^5^ consider the common resource to be a dynamically renewable system that is also influenced by a feedback of strategy of players. Such interdependence is modelled by a co-evolutionary system where both strategy and resource are subject to change.

When we focus on the study of human behaviour, scientists enthusiastically discuss whether human deliberation promotes or inhibits cooperation. There is no decisive knowledge regarding which viewpoint is correct. According to Grossmann *et al*.^6^, humans are expected to cooperate intuitively^7^, on the other hand, they do deliberative behaviour, i.e. social comparison^8^, self-reflection^9^, and mental simulation of the future^10^. Social comparison means that people evaluate their own opinions and abilities and define themselves. Humans utilize self-reflection to infer the mental state of others and perform mental simulation of the future to predict a pleasant result which has never been experienced before. One study^11^ states that such deliberative behaviour (hereinafter referred to as deliberation) has a positive correlation with cooperation, while other studies^12,13^ describe that deliberation hinders cooperation in the situation of social dilemma. Some studies^14–16^, which refer to the nature of deliberation, show that wise deliberation, i.e. capturing the situation with larger perspective including sensitivity to temporal and social dependence between incidents, integrates the purpose of defence and cooperation, thereby humans can maintain cooperation. Regarding the time of deliberation, Yamagishi *et al*.^17^ show that altruists spending shorter time and egoists spending longer time in decision-making will cooperate with each other.

Based on those results of studies of human behaviour, in the study of evolutionary game theory, models considering the cost of deliberation are increasing. Bear and Rand^18^ introduce the mechanism that when players play both one-shot and reciprocal prisoner’s dilemma game, they pay the (stochastically fluctuating) cost and adapt their strategy to the type of game they are facing. As the probability of reciprocity of the prisoner’s dilemma game gets higher, ‘dual-process cooperators’ that cooperate intuitively, but consider defecting to maximize their payoff will become dominant instead of defectors who do not deliberate. Based on that study, Mosleh and Rand^19^ extend the model of Bear and Rand^18^ and find that regardless of the type of topology of connections, sparser (i.e. lower average degree) network lowers the level of deliberation of such dual-process cooperators, while it promotes the success of dual-process cooperators against intuitive defectors.

Here, based on the trend of evolutionary game theory, this study considers that the situation where a person must make his/her decision forces a psychological burden on him/her. The extent of his/her psychological burden changes depending on whether he/she requires only a short time or needs a long time when he/she makes his/her decision. If he/she needs only a short time, such burden is light, and if he/she needs a long time, such burden is heavy. This study defines such burden as the cost of decision. As shown by Bear and Rand^18^, the concept of costly decision is based on the results of the studies regarding human behaviour^20,21^.

This study utilizes the model of evolutionary game theory that firstly, each player plays the spatial prisoner’s dilemma game with opponent players who are connected to him/her, and secondly, he/she makes his/her decision with paying the cost. As described before, a player with high cost of decision has a property that he/she requires a long time in decision, and a player with low cost of decision has a property that he/she needs only a short time in decision. While the cost of deliberation proposed by Bear and Rand^18^ fluctuates stochastically in decision, the cost of decision of this study is a random value in the range [0, 1] that is initially assigned to each player. The references^18,19^ allow players to sense whether the game is repeated or not, however, this study does not permit players to recognise such information. Those are the major differences between the references^18,19^ and this study. When each player copies the strategy of the player who is connected to him/her and has the highest payoff, he/she also imitates the cost of decision of that player; i.e. he/she imitates not only the strategy but also the property of the most successful neighbour player.

This study explores (1) whether cooperation evolves or not and (2) whether players with low cost (i.e. property of fast decision) or players with high cost (i.e. property of slow decision) become mainstream as a result of coevolutionary dynamics between the cost of decision and the strategy in various types of sparse topology of connections. The main result of this study is that the introduction of the cost of decision contributes to the evolution of cooperation, although there are some differences in the extent of its contribution regarding the three types of sparse topology of connections. Regarding the distribution of the cost of decision, especially in the case of the scale-free topology of connections, players with low cost of decision are not necessarily mainstream, and players with high cost of decision are sometimes mainstream at the last.

## Model

**Figure 1:**
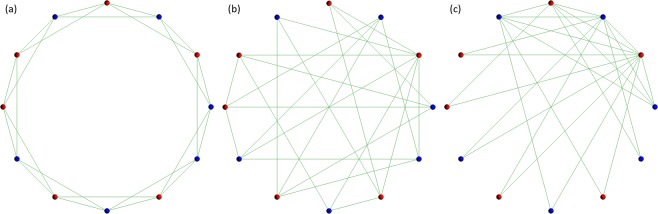
This figure is Fig. 1 of the previous study^26^. Three panels (a), (b), and (c) show the (**a**) regular, (**b**) random, and (**c**) scale free topology of connections in the case of <*k*> = 4. Each topology is defined as one dimensional lattices of periodic boundary conditions, and a vertex represents a player. Cooperators are shown in blue, and defectors are shown in red. Although the model of this study has *N* = 1000 players, this figure has only 12 players to make each topology easy to understand.

The model of this study is based on the agent-based model of the previous studies^22,23^. As shown in Fig. 1, it deals with the three types of topology of connections, i.e. (a) regular^24^, (b) randomly rewired (random)^24^, (c) the case where limited players have quite a lot of connections (scale-free)^25^. Each topology is one-dimensional lattices of periodic boundary conditions, and each vertex represents each player. The degree of player *i* (the number of connections) is *k*(*i*), and this study deals with the case where the average of *k*(*i*), i.e.$< k > =\frac{1}{N}\sum _{1\le i\le N}k(i)=4$. The detail of how to construct each topology is described in the previous study^22^. Figure 1 shows the three types of topology of connections in the case of <*k*> = 4. In the model, the number of players *N* equals 1000, however, this figure sets *N* = 12 to make each topology easy to understand.

The strategy of player *i* (*s*(*i*)) is the two types, i.e. either defection or cooperation, and is expressed as (0 1) or (1 0) utilizing unit vectors, respectively. Player *i* plays the prisoner’s dilemma game with all opponent players connected to him/her and obtains total payoff *P*(*i*). When *j* is the opponent player of player *i*, and *s*(*j*) is the strategy of player *j*, *P*(*i*) is expressed by the following Equation (1) utilizing the payoff matrix *A*. The parameter *b* in the payoff matrix *A* equals 1.5 in the model. *O*(*i*) represents the set of all players connected to player *i*. In the initial state, the ratio of the number of players of defection (defectors) to the number of players of cooperation (cooperators) is almost one to one. At the start of each simulation run, defectors and cooperators are randomly distributed.

1 $$\begin{array}{c}P(i)=\sum _{j\in O(i)}s(i)As{(j)}^{T},A=(\begin{array}{cc}1 & 0\\ b & 0\end{array})\,\,\,\,\,\,\,\,(1 < b\le 2)\\ (i\ne j,\,\,\,1\le i,\,\,j\le N)\end{array}$$

After all players have finished playing the prisoner’s dilemma game with opponent players, firstly, player *i* pays the cost of decision (*D*(*i*)) that is a random initial value in the range of [0, 1] in decision. As a result, the payoff of player *i* (*P*(*i*)) changes as shown in the following Equation (2).

2 $$P(i)\text{'}=P(i)-D(i)$$

Secondly, as the following Equation (3), player *i* imitates the strategy of player *j*_max_ $\in i\cup O(i)$ and adopts it as his/her strategy in the matches of the next generation. At the same time, he/she also imitates the cost of decision *D*(*j*_max_) of player *j*_max_. When there are multiple candidates for player *j*_max_, player *i* randomly selects his/her strategy and cost of decision from the strategy and the cost of decision of those candidates in the matches of the next generation. The process of adopting new strategy and cost of decision is executed simultaneously regarding all players.

3 $$\begin{array}{c}s{(i)}^{\text{'}}=s({j}_{\max })\,\,{j}_{\max }\in i\cup O(i)\\ D{(i)}^{\text{'}}=D({j}_{\max })\,\,{j}_{\max }\in i\cup O(i)\\ P{({j}_{\max })}^{\text{'}}=\,\max ({P}^{\text{'}}\in i\cup O(i))\end{array}$$

This study defines all matches of the prisoner’s dilemma game and adoption of new strategy with the cost of decision regarding all players as one generation, and each simulation run continues until the number of generations reaches 300. The following results are the average of 20 simulation runs and have error bars indicating standard errors (SE) if necessary. The reason why this study employs SE is that we are interested in how well the true distribution is being estimated by the multiple replicates. Except in the Discussion, basically the author rounds each value off to three decimal places.

## Results

**Figure 2:**
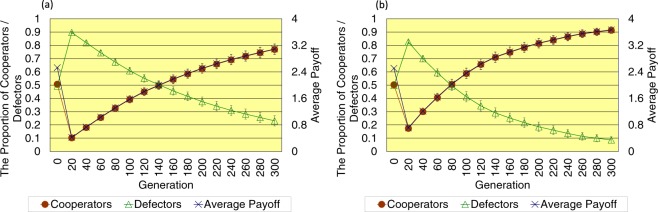
This figure shows the time series results of the proportion of cooperators (left vertical axis), the proportion of defectors (left vertical axis), and the average payoff of all players (right vertical axis) regarding the regular topology of connections (**a**) without/(**b**) with the cost of decision (error bars: SE, standard errors).

Firstly, the author describes the proportion of cooperators, the proportion of defectors, and the average payoff of all players in the last 300 generation. As shown in Fig. 2, when the topology of connections is regular, in the case without the cost of decision, the proportion of cooperators is 0.769 ± 0.039 (SE), the proportion of defectors is 0.231 ± 0.039 (SE), and the average payoff of all players is 3.089 ± 0.155 (SE) in the last 300 generation. On the other hand, in the case with the cost of decision, the proportion of cooperators is 0.913 ± 0.024 (SE), the proportion of defectors is 0.087 ± 0.024 (SE), and the average payoff of all players is 3.666 ± 0.098 (SE) in the last 300 generation. When introducing the cost of decision, the proportion of cooperators increases, the proportion of defectors decreases, the average payoff of all players increases in the last 300 generation, and the dispersion of each value decreases.

Regarding the stability of the evolutionary state, the author shows another result of the evolution of cooperation in the case where the topology of connections is regular, and the number of generations is 600 in the Supplementary Information (see Fig. S3). This additional result shows that the evolution of cooperation occurs regardless of the introduction of the cost of decision. However, it also indicates that the introduction of the cost of decision apparently promotes the evolution of cooperation because in the case with the cost of decision, the proportion of cooperators exceeds 0.9, and the proportion of defectors is less than 0.1 within approximately a half (i.e. 300) generation in comparison with the case without the cost of decision. Therefore, with the results until 300 generations, we can sufficiently grasp the evolution of cooperation by the introduction of the cost of decision.

**Figure 3:**
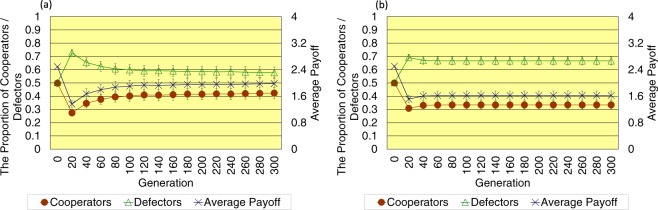
This figure shows the time series results of the proportion of cooperators (left vertical axis), the proportion of defectors (left vertical axis), and the average payoff of all players (right vertical axis) regarding the random topology of connections (**a**) without/(**b**) with the cost of decision (error bars: SE, standard errors).

As shown in Fig. 3, when the topology of connections is random, in the case without the cost of decision, the proportion of cooperators is 0.423 ± 0.043 (SE), the proportion of defectors is 0.577 ± 0.043 (SE), and the average payoff of all players is 1.981 ± 0.189 (SE) in the last 300 generation. On the other hand, in the case with the cost of decision, the proportion of cooperators is 0.334 ± 0.030 (SE), the proportion of defectors is 0.666 ± 0.030 (SE), and the average payoff of all players is 1.620 ± 0.135 (SE) in the last 300 generation. When introducing the cost of decision, the proportion of cooperators slightly decreases, the proportion of defectors slightly increases, and the average payoff of all players decreases a little in the last 300 generation, while the dispersion of each value decreases as well as the results of the regular topology of connections.

**Figure 4:**
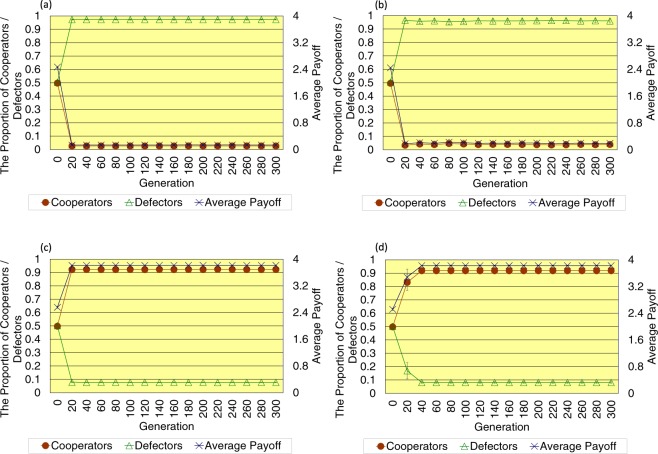
This figure shows the time series results of the proportion of cooperators (left vertical axis), the proportion of defectors (left vertical axis), and the average payoff of all players (right vertical axis) regarding the scale-free topology of connections (**a**,**c**) without/(**b**,**d**) with the cost of decision (error bars: SE, standard errors). The upper row indicates the results that defectors become dominant, while the lower row depicts the results that cooperators become dominant.

As shown in Fig. 4, when the topology of connections is scale-free, there occurs some simulation runs that defectors become dominant in the last 300 generation. The number of such simulation runs is 14 out of 20 in the case without the cost of decision, whereas it decreases to 10 out of 20 in the case with the cost of decision. Especially regarding the results that cooperators become dominant in the last 300 generation, in the case without the cost of decision, the proportion of cooperators is 0.9225 ± 0.002 (SE), the proportion of defectors is 0.0775 ± 0.002 (SE), and the average payoff of all players is 3.815 ± 0.005 (SE). On the other hand, in the case with the cost of decision, the proportion of cooperators is 0.918 ± 0.006 (SE), the proportion of defectors is 0.082 ± 0.006 (SE), and the average payoff of all players is 3.825 ± 0.013 (SE). When introducing the cost of decision, the proportion of cooperators, the proportion of defectors, and the average payoff of all players in the last 300 generation hardly change, while the dispersion of each value slightly increases as opposed to the results of the regular and random topology of connections.

**Figure 5:**
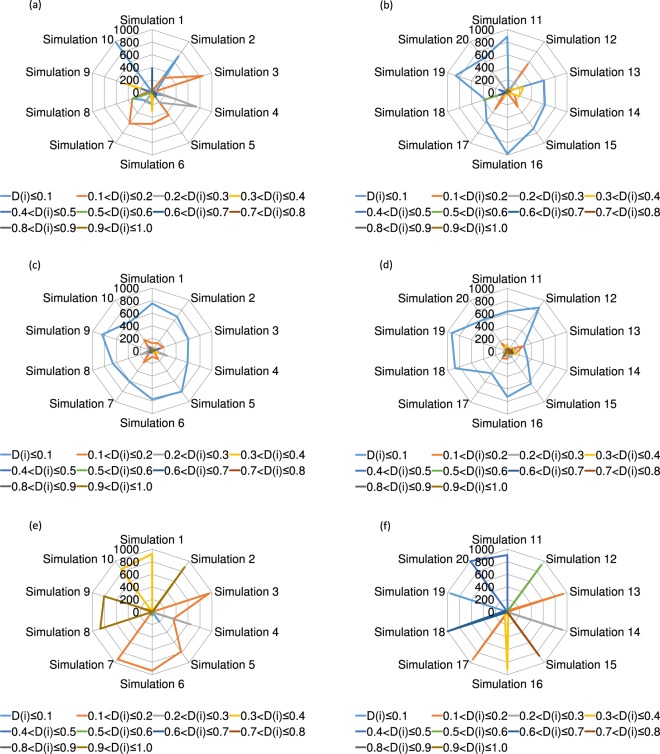
This figure shows how many players have the cost of decision (*D*(*i*)) of each range when the topology of connections is regular (**a**,**b**), random (**c**,**d**), and scale-free (**e**,**f**). Panels of the left column (**a**,**c**,**e**) indicate the results of Simulations from 1 to 10, and those of the right column (**b**,**d**,**f**) depict the results of Simulations from 11 to 20. When the topology of connections is scale-free (**e**,**f**), defectors-dominant simulation runs in the last 300 generation are Simulations 1, 2, 4, 5, 9, 10, 13, 14, 18, and 20, while cooperators-dominant simulation runs in the last 300 generation are Simulations 3, 6, 7, 8, 11, 12, 15, 16, 17, and 19.

Secondly, the author describes how many players have the cost of decision that is less than or equal to 0.5 (i.e. *D*(*i*) ≤ 0.5) in the last 300 generation. As shown in Fig. 5, when the topology of connections is regular, the number of players with *D*(*i*) ≤ 0.5 is 900.65 ± 38.784 (SE), and when it is random, such number is 928.5 ± 16.48 (SE). Therefore, in both cases, the number of players with *D*(*i*) ≤ 0.5 amounts to nearly 90 percent of the number of players *N* on average. When comparing those two cases, we can find that the number of players with *D*(*i*) ≤ 0.5 is smaller, and the dispersion of such number is larger when the topology of connections is regular than when the topology of connections is random. On the other hand, as also shown in Fig. 5, when the topology of connections is scale-free, the number of players with *D*(*i*) ≤ 0.5 is 711.2 ± 95.167 (SE), and such number is small and quite dispersed in comparison with each number regarding the regular and random topology of connections. Especially, the number of players with 0.9 < *D*(*i*) ≤ 1.0, the number of players with 0.5 < *D*(*i*) ≤ 0.6, the number of players with 0.7 < *D*(*i*) ≤ 0.8, and the number of players with 0.6 < *D*(*i*) ≤ 0.7 is the largest in all ranges of *D*(*i*) in Simulations 2, 8, 9, Simulation 12, Simulation 15, and Simulation 18, respectively.

## Discussion

Firstly, the author discusses the effect of the introduction of the cost of decision on the proportion of cooperators, the proportion of defectors, and the average payoff of all players in the last 300 generation when the topology of connections is regular and random. The author refers to that effect regarding the scale-free topology of connections in the discussion regarding the results of the number of players with *D*(*i*) ≤ 0.5 in the last 300 generation.

**Figure 6:**
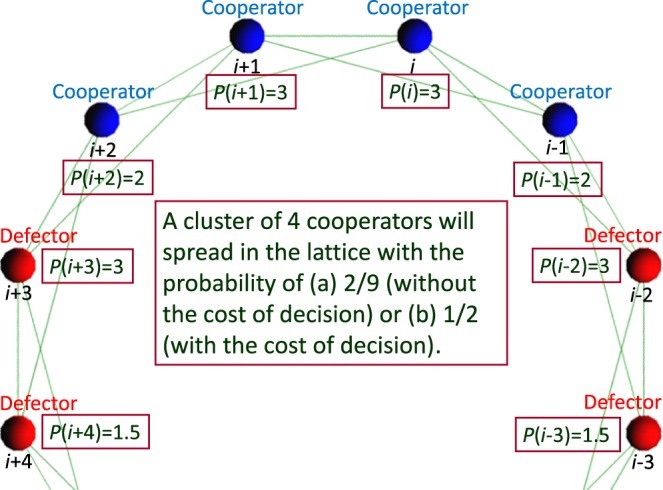
This figure explains the probability that a cluster of 4 cooperators will spread regarding both cases (a) without/(b) with the cost of decision.

When the topology of connections is regular, the introduction of the cost of decision clearly contributes to the evolution of cooperation. This is because as shown in Fig. 6, the probability that a cluster of 4 cooperators will spread further is 2/9 when the cost of decision is not introduced, whereas such probability increases to 1/2 when introducing the cost of decision. For each case, the calculation method of such probability is as follows. When the cost of decision is not introduced, because the probability that each defector (*i* − 2, *i* + 3) adjacent to a cluster of 4 cooperators will be a cooperator is 1/2, and the probability that the cooperators on the boundary of the same cluster (*i* − 1, *i* + 2) and the cooperators in the centre (*i*, *i* + 1) remain as cooperators is the product of 2/3 and 2/3 (=4/9), we can find the probability that a cluster of 4 cooperators will spread further as the product of 1/2 and 4/9 (=2/9). On the other hand, when introducing the cost of decision, in the case where each defector (*i* − 2, *i* + 3) adjacent to a cluster of 4 cooperators has greater cost of decision than each cooperator (*i*, *i* + 1), such defector becomes a cooperator with the probability of 1/2, respectively. At that time, each cooperator (*i* − 1, *i* + 2) on the boundary of the same cluster and each cooperator (*i*, *i* + 1) in the centre remain as cooperators. Here, the probability that each defector (*i* − 2, *i* + 3) adjacent to a cluster of 4 cooperators has the same cost of decision as each cooperator (*i*, *i* + 1) is extremely small (1/32767) and then can be ignored.

When the topology of connections is random, the introduction of the cost of decision does not contribute much to the evolution of cooperation. When comparing both cases where the topology of connections is regular and random, the clustering coefficient is high in the regular case, that is, a cluster of cooperators is more likely to be constructed in the regular case. On the other hand, when the topology of connections is random, because the average path length is short in comparison with the regular case, and there are some connections between players with long distance, clusters of cooperators are easily exploited by defectors away from such clusters, and then such clusters are difficult to spread. Under those circumstances, the cost of decision randomly allocated regardless of the strategy of each player has the effect of suppressing the dispersion of the proportion of cooperators, the proportion of defectors, and the average payoff of all players, but it does not clearly enhance the evolution of cooperation.

Secondly, the author discusses the results of the number of players with *D*(*i*) ≤ 0.5 in the last 300 generation. As described in the Results, when comparing both cases where the topology of connections is regular and random, the number of players with *D*(*i*) ≤ 0.5 is smaller, and the dispersion of such number is larger in the regular case than in the random case. The reason why such results occur is that in the regular case, the number of players with *D*(*i*) ≤ 0.5 becomes small in the following two cases (see Table 1). The first one is the case where the proportion of defectors becomes extremely large in the initial 30 generations, and the proportion of cooperators does not increase sufficiently in 300 generations (Simulations 1 and 5). The second one is the case where the proportion of defectors increases only in the initial 10 generations, and then the proportion of cooperators increases rapidly (Simulations 8 and 18). As for the first one, because the proportion of cooperators does not increase sufficiently, the cost of decision that each player possesses also fluctuates and does not converge. As for the second one, because the proportion of cooperators increases rapidly, large clusters of cooperators are built quickly, and players with the cost of decision slightly exceeding 0.5 can become mainstream in such clusters. More specifically, in Simulations 1 and 5, the number of generations where the proportion of cooperators becomes above 0.1 is 32 and 29, respectively. On the other hand, in Simulations 8 and 18, such number of generations is 9 and 8, respectively. In Simulations 8 and 18, the cost of decision *D*(*i*) that players most possess is 0.511704 and 0.515458, respectively. In addition, even in the random case, because there are some cases where players with a lot of connections relatively have high payoff and may not be disadvantageous even if they have high *D*(*i*), as in Simulations 13, 14, and 17, the cost of decision that each player possesses is sometimes maintained relatively dispersed.

**Table 1:** This table shows the results of the number of simulation runs, the proportion of cooperators, the proportion of defectors, and the average payoff of all players regarding the regular topology of connections in the case of the relatively small number of players with *D*(*i*) ≤ 0.5.

The number of simulation runs	The proportion of cooperators	The proportion of defectors	Average payoff
Simulation 1	0.682	0.318	2.734
Simulation 5	0.6	0.4	2.408
Simulation 8	0.951	0.049	3.819
Simulation 18	0.957	0.043	3.844

When the topology of connections is scale-free, the clustering coefficient is higher, and it is easier for cooperators to construct clusters than when the topology of connections is random. However, because the average path length is shorter than in the regular case, and there are some connections between distant players, clusters of cooperators are likely to be exploited by defectors away from such clusters. Therefore, the introduction of the cost of decision contributes to the evolution of cooperation in the scale-free case rather than in the random case, while it cannot stably contribute to the evolution of cooperation in comparison with the regular case.

More specifically, when the topology of connections is scale-free, the degree of each player (*k*(*i*)) is distributed over a wider range than when the topology of connections is random. In addition, because each player so-called “hub” of high degree has considerably high payoff, the payoff of such player is not affected so much by the cost of decision (*D*(*i*)) of relatively small value (0 ≤ *D*(*i*) ≤ 1). Therefore, in the last 300 generation, players with low cost of decision are not necessarily mainstream, and players with high cost of decision may be mainstream. However, as Simulations 8 and 15, when cooperators become dominant in the last 300 generation, and players with high cost of decision become mainstream, the proportion of cooperators and the average payoff of all players are somewhat reduced, and on the contrary, the proportion of defectors somewhat increases in comparison with each average value described in the Results (see Table 2). This is the reason why in the scale-free case introducing the cost of decision, the dispersion of the proportion of cooperators, the proportion of defectors, and the average payoff of all players increases a little when cooperators become dominant in the last 300 generation.

**Table 2:** This table shows the results of the number of simulation runs, the proportion of cooperators, the proportion of defectors, and the average payoff of all players regarding the scale-free topology of connections in the case where cooperators become dominant, and players with high cost of decision are mainstream in the last 300 generation.

The number of simulation runs	The proportion of cooperators	The proportion of defectors	Average payoff
Simulation 8	0.895	0.105	3.796
Simulation 15	0.859	0.141	3.6785

In this study, based on the studies of evolutionary game theory^18,19^, the author considers that the situation where a person must make his/her decision forces a psychological burden on him/her and defines such burden as the cost of decision. Then, this study proposes the model that firstly, each player plays the spatial prisoner’s dilemma game with opponent players connected to him/her, and secondly, he/she makes his/her decision with paying the cost. In this model, when each player copies the strategy of the player who is connected to him/her and has the highest payoff, he/she also imitates the cost of decision of that player. The results of that coevolutionary dynamics show the following facts regarding the three types of sparse topology of connections (<*k*> = 4).

1. When the topology of connections is regular, the introduction of the cost of decision clearly contributes to the evolution of cooperation because in the case with the cost of decision, over 90 percent of players become cooperators within approximately a half (i.e. 300) generation in comparison with the case without the cost of decision.
2. When the topology of connections is random, the introduction of the cost of decision does not contribute much to the evolution of cooperation, while it suppresses the dispersion of the proportion of cooperators, the proportion of defectors, and the average payoff of all players.
3. When the topology of connections is scale-free, the introduction of the cost of decision contributes to the evolution of cooperation rather than when the topology of connections is random, while it cannot stably contribute to the evolution of cooperation in comparison with the regular case.

The above results show that the introduction of the cost of decision contributes to the evolution of cooperation, although there are some differences in the extent of its contribution regarding the three types of sparse topology of connections. That knowledge resembles the result described by Mosleh and Rand^19^. On the other hand, they also describe that the level of deliberation commonly decreases in different types of sparse topology of connections, however, such phenomenon is not observed in the results of this study. In other words, in this study, when the topology of connections is scale-free, players with low cost of decision are not necessarily mainstream, and players with high cost of decision are sometimes mainstream in the last 300 generation. This is because players of high degree have considerably high payoff, and the payoff of such players is not affected so much by the cost of decision (*D*(*i*)) of relatively small value (0 ≤ *D*(*i*) ≤ 1).

In the future works, it will be necessary to investigate whether there is a value of the cost of decision that can contribute to the evolution of cooperation in the case of <*k*> = 8, 16. The investigation of the evolution of cooperation in the case of another topology of connections (e.g. small world and star networks) is also required. As the preliminary experiment, the author shows the result of the evolution of cooperation in the small world network (rewired with the probability *p* = 0.1) in the Supplementary Information (see Fig. S4). Adding a small number of shortcuts to the regular topology of connections favours defectors, however, the introduction of the cost of decision is effective in suppressing that advantage, and further analysis is necessary for the distribution of the cost of decision. Regarding the star network, in the case of the simple centralized topology, we can easily guess that the result of evolutionary dynamics depends on the strategy of the central player in the initial state, however, further analysis is required for the topology with the tree structure. In addition, like Ohdaira’s study^26^, the author plans to introduce actions of players removing and creating connections based on their preferences into the model of this study, and to investigate whether such actions can lead to the further evolution of cooperation.

## Supplementary information

Supplementary Information
