# Supplementary material for: Coevolution between the cost of decision and the strategy contributes to the evolution of cooperation
Source: Sci Rep. 2019 Mar 14;9:4465. doi: 10.1038/s41598-019-41073-9 (PMC6418178; doi:10.1038/s41598-019-41073-9)
Supplement: Supplementary file 1 — Supplementary Information [file 41598_2019_41073_MOESM1_ESM.pdf]

Supplementary Information: Coevolution between the cost of decision and the strategy contributes to the evolution of cooperation

Tetsushi Ohdaira†

†*Institute of Information and Media, Aoyama Gakuin University, 5-10-1 Fuchinobe, Chuo-ku, Sagamihara-city, Kanagawa 252-5258 Japan*

In this Supplementary Information, to contribute to the comprehension of the paper, the author shows following figures; i.e. (1) the degree distribution (the probability distribution of degrees) of the regular, random, and scale-free topology of connections of $\langle k \rangle=4$ in the initial state (Figure S1), (2) another type of charts (i.e. three-dimensional area charts) indicating how many players have the cost of decision ($D(i)$) of each range regarding the three types of topology of connections (Figures S2(a, b), S2(c, d), and S2(e, f)), (3) another result of the evolution of cooperation in the case where the topology of connections is regular, and the number of generations is 600 (Figure S3), and (4) the result of the evolution of cooperation in the small world network (rewired with the probability p=0.1) as the preliminary experiment (Figure S4).

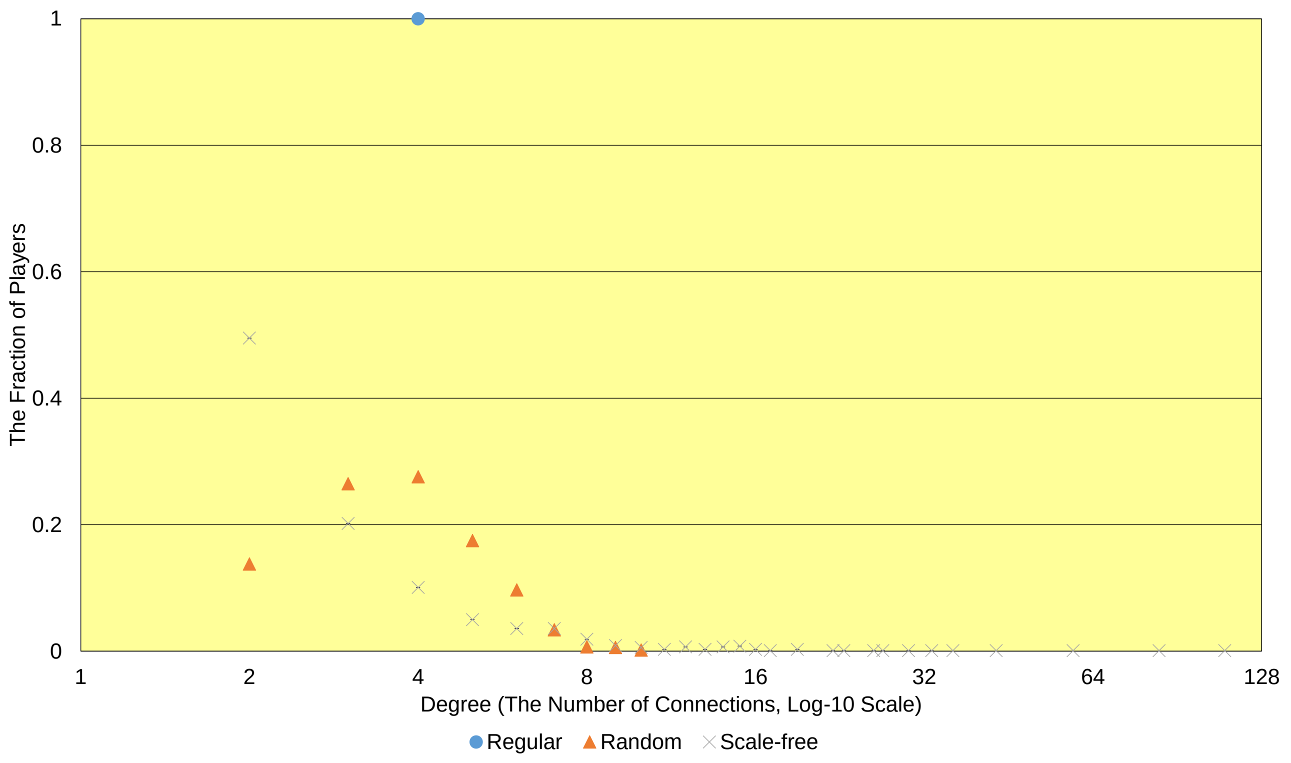

Figure S1. This figure depicts the degree distribution of the regular, random, and scale-free topology of connections of <*k*>=4 in the initial state. The horizontal axis with log-10 scale indicates the degree of a player in a lattice, i.e. his/her number of connections to other players. The vertical axis exhibits the fraction of players in a lattice with each degree.

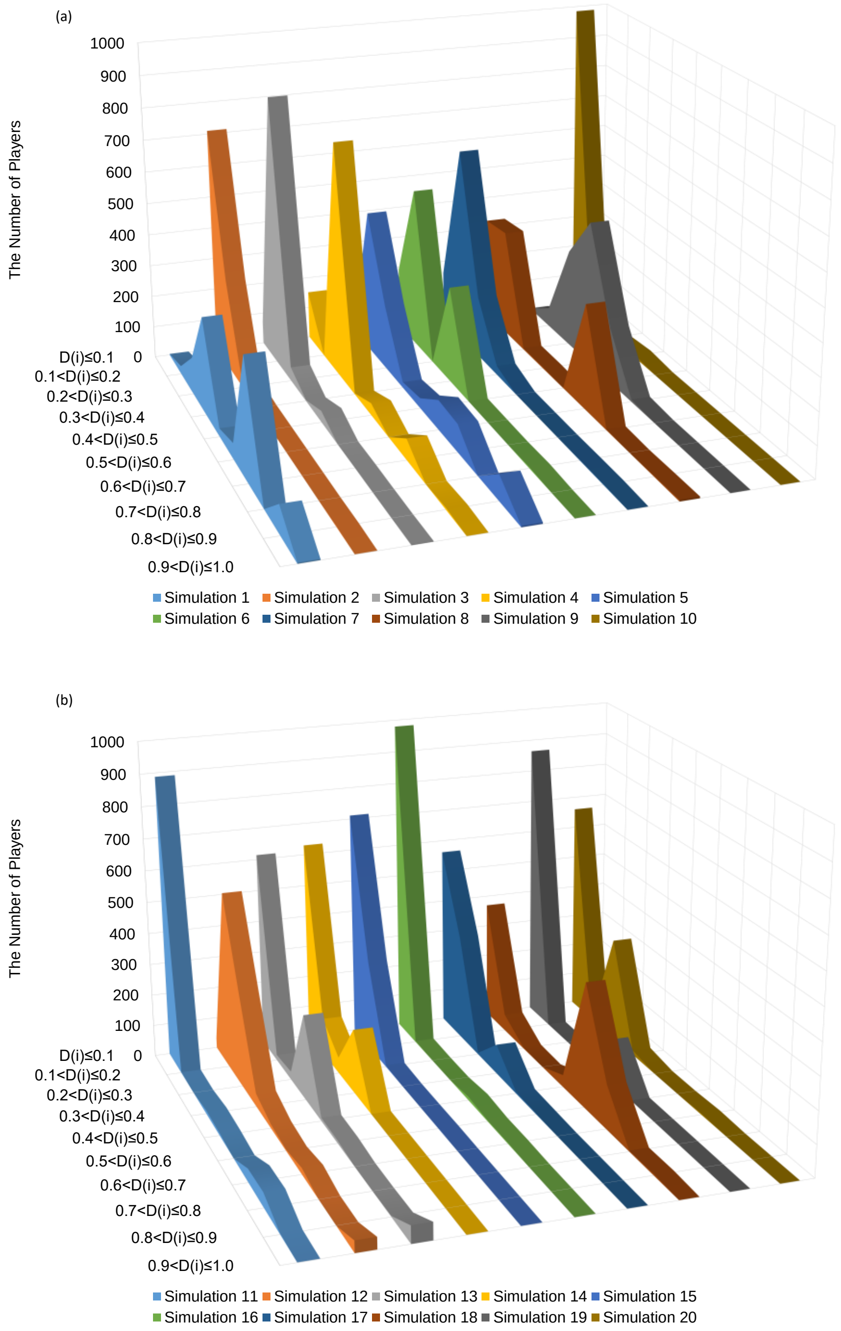

Figure S2(a, b). These three-dimensional area charts indicate how many players have the cost of decision ($D(i)$) of each range when the topology of connections is regular. The upper panel (**a**) indicates the results of Simulations from 1 to 10, and the lower panel (**b**) depicts the results of Simulations from 11 to 20.

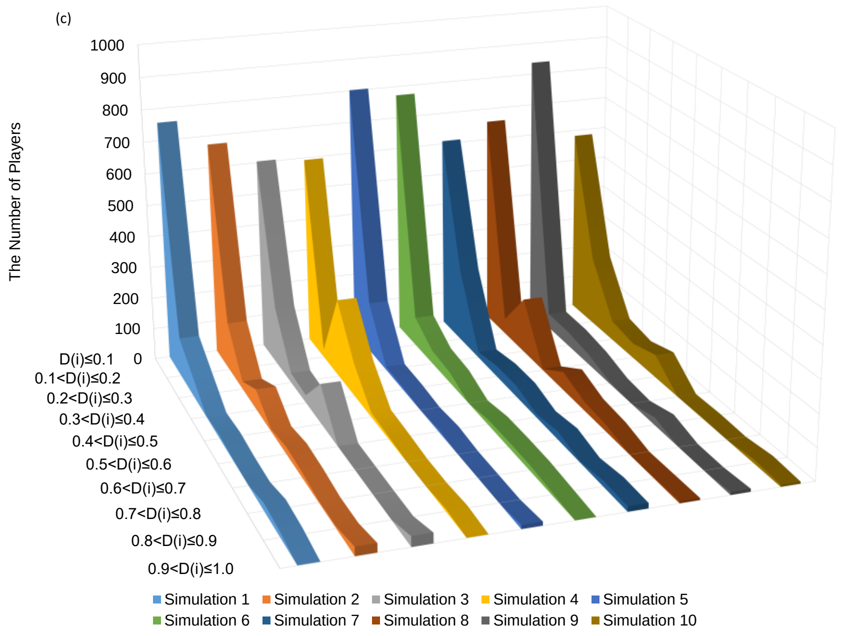

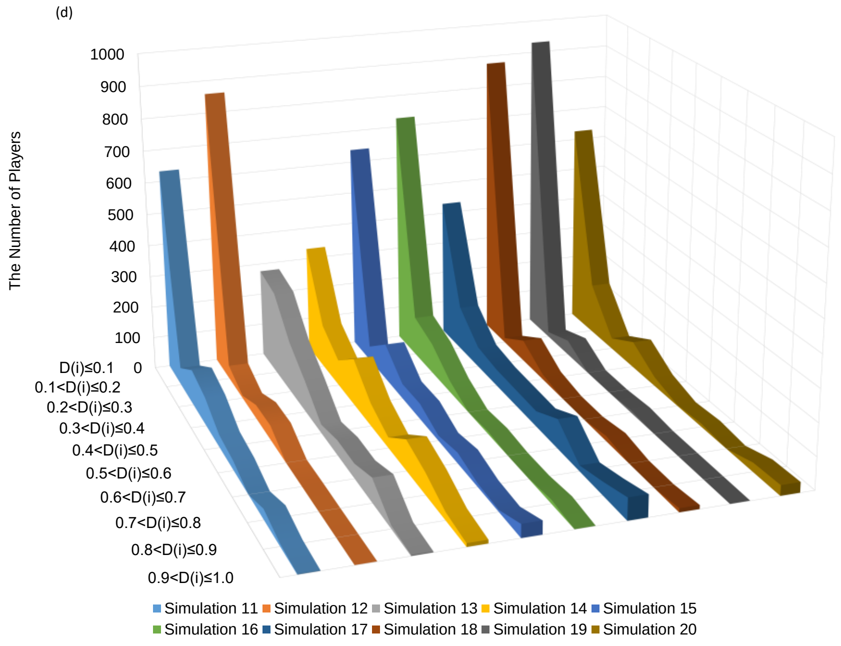

Figure S2(c, d). These three-dimensional area charts indicate how many players have the cost of decision ($D(i)$) of each range when the topology of connections is random. The upper panel (**c**) indicates the results of Simulations from 1 to 10, and the lower panel (**d**) depicts the results of Simulations from 11 to 20.

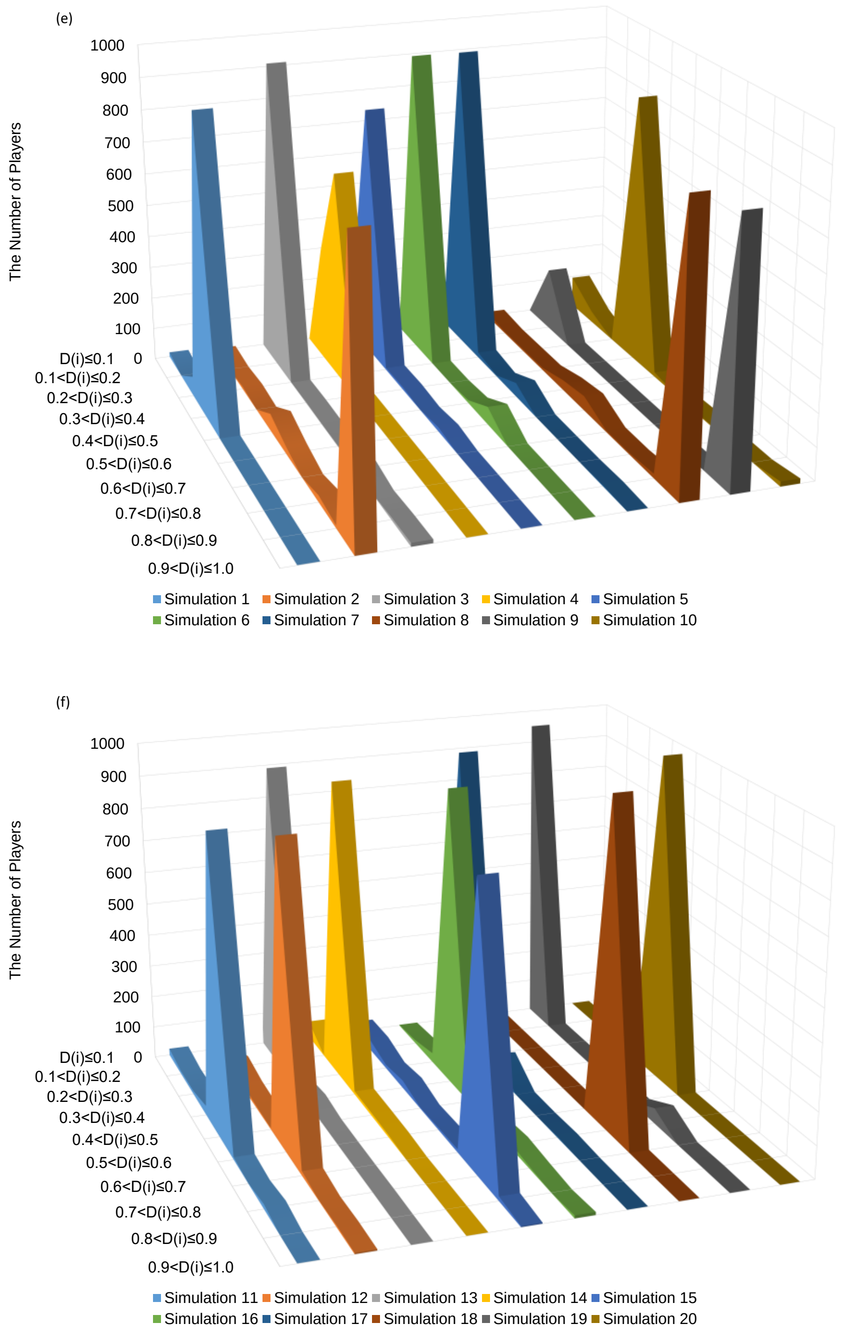

Figure S2(e, f). These three-dimensional area charts indicate how many players have the cost of decision ($D(i)$) of each range when the topology of connections is scale-free. The upper panel (**e**) indicates the results of Simulations from 1 to 10, and the lower panel (**f**) depicts the results of Simulations from 11 to 20. Note that defectors-dominant simulation runs in the last 300 generation are Simulations 1, 2, 4, 5, 9, 10, 13, 14, 18, and 20, while cooperators-dominant simulation runs in the last 300 generation are Simulations 3, 6, 7, 8, 11, 12, 15, 16, 17, and 19.

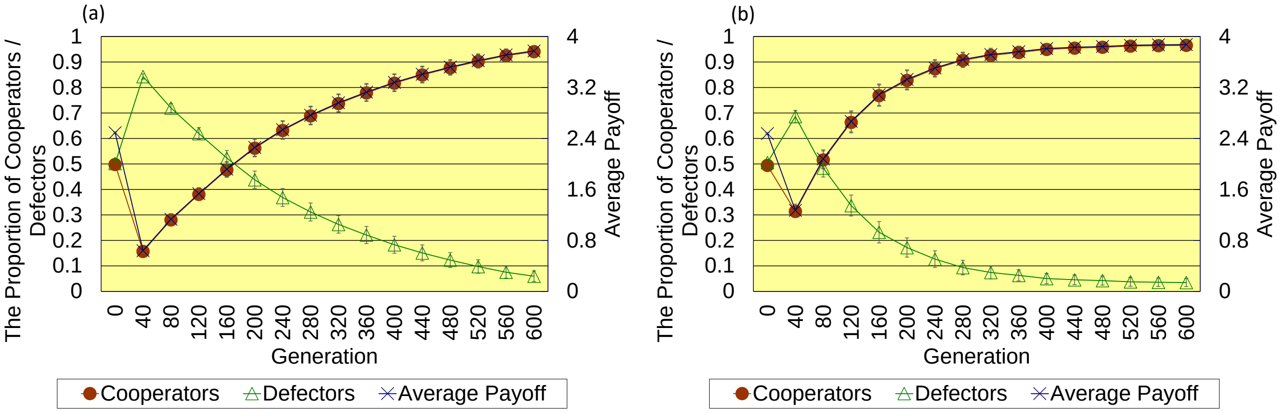

Figure S3. This figure shows the time series results of the proportion of cooperators (left vertical axis), the proportion of defectors (left vertical axis), and the average payoff of all players (right vertical axis) regarding the regular topology of connections (a) without / (b) with the cost of decision in 600 generations (error bars: SE, standard errors).

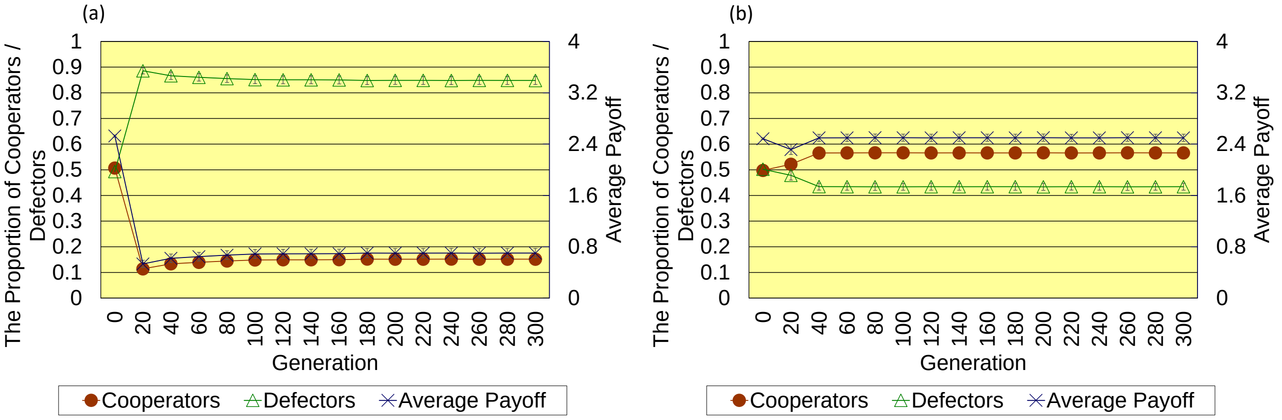

Figure S4. This figure shows the time series results of the proportion of cooperators (left vertical axis), the proportion of defectors (left vertical axis), and the average payoff of all players (right vertical axis) regarding the small world network (rewired with the probability p=0.1) (a) without / (b) with the cost of decision in 300 generations (error bars: SE, standard errors).
